# Prevalence of *Cryptosporidium* infection and associated risk factors in calves in Egypt

**DOI:** 10.1038/s41598-023-44434-7

**Published:** 2023-10-18

**Authors:** Hattan S. Gattan, Ayed Alshammari, Mohamed Marzok, Mohamed Salem, Omar A. AL-Jabr, Abdelfattah Selim

**Affiliations:** 1 Department of Medical Laboratory Sciences, Faculty of Applied Medical Sciences, King Abdulaziz University, Jeddah, Saudi Arabia; 2 Special Infectious Agents Unit, King Fahad Medical Research Center, King AbdulAziz University, Jeddah, Saudi Arabia; 3 Department of Biology, College of Science, University of Hafr Al-Batin, Hafr Al-Batin, Saudi Arabia; 4 Department of Clinical Sciences, College of Veterinary Medicine, King Faisal University, 31982 Al-Ahsa, Saudi Arabia; 5 Department of Surgery, Faculty of Veterinary Medicine, Kafr El Sheikh University, Kafr El Sheikh, Egypt; 6 Department of Medicine and Infectious Diseases, Faculty of Veterinary Medicine, Cairo University, Cairo, 12613 Egypt; 7 Department of Microbiology, College of Veterinary Medicine, King Faisal University, P.O. Box 400, 31982 Al-Ahsa, Saudi Arabia; 8 Department of Animal Medicine (Infectious Diseases), Faculty of Veterinary Medicine, Benha University, Toukh, 13736 Egypt

**Keywords:** Parasitology, Risk factors

## Abstract

*Cryptosporidium* is one of the causative parasitic agents that causes gastrointestinal diseases in calves. The parasite poses a zoonotic risk to immunocompromised individuals and children. Thus, this study aimed to determine the prevalence of *Cryptosporidium* infection in calves in three Egyptian governorates situated in Nile Delta and assess the associated risk factors. The *Cryptosporidium* oocysts were detected in 81 out of 430 calves (18.84%). In addition, the univariant analysis showed that age, feeding source, hygienic status, presence of diarrhea and contact with other animals were significantly (*P* < 0.05) associated with *Cryptosporidium* prevalence in calves. Furthermore, the risk factors related with *Cryptosporidium* prevalence were age (OR 1.96, 95%CI 0.97–3.94), feeding on milk and pasture (OR 2.07, 95%CI 1.15–3.72), poor hygienic condition (OR 2.25, 95%CI 1.28–3.94), presence of diarrhea (OR 2.47, 95%CI 1.23–4.96) and contact with other domestic animals (OR 2.08, 95%CI 1.24–3.50). In addition, the PCR assay targeting 18srRNA showed that the most prevalent species among calves was *C. parvum*. Although additional researches are required to understand the most effective steps that farmers and veterinary professionals should take to decrease the occurrence of *Cryptosporidium* infection.

## Introduction

*Cryptosporidium* is an intracellular protozoan and one of the most common enteric pathogens in claves during first two weeks of life. Four species are usually discovered infecting cattle: *C. parvum, C. andersoni, C. bovis*, and *C. ryanae*^1,2^. *C. parvum* is commonly associated with diarrhoea in susceptible hosts, causing sickness and even mortality, notably in neonatal calves^3^. The life cycle of this pathogen is direct, and it can grow and replicate in infected animal's gastrointestinal epithelial cells^4,5^. The infective stage of *Cryptospordium*'s life cycle is the oocyst, which is secreted in the faces and contains four sporozoites. When the oocyst is ingested, sporozoites are released. These sporozoites enter the cells, forming oocysts with thick and thin walls. The thick-walled oocyst is discharged in the faces. The thin-walled oocysts can rupture, allowing the sporozoites to infect new host enterocytes and produce autoinfection, leading to relapses or persistent gut illness. These sporozoites go through several phases before creating new oocysts. Cell infection results in cell death, which causes intestinal villi to shrink and fuse^6^.

In addition, the parasite can be passed from person to person, animal to animal, or animal to human (zoonotic transmission)^7^.

Infections are usually transmitted through the faecal-oral route, with infective stages of expelled sporulated oocysts through direct or collateral contact^8^. The infection is known to be self-limiting in the immunocompetent hosts, but it can cause acute or severe diarrhea in young animals or in immunocompromised hosts^9^.

Even though bovine cryptosporidiosis has been identified as a significant contributor to newborn diarrhea and financial losses on dairy farms, it is frequently misdiagnosed^10^. It is characterized by anorexia, abdominal pain and diarrhea, which can cause slow growth and even death. Diarrhea usually begins 3–5 days after infection and lasts 4 to 17 days in most of infected calves^11^. Oocyst shedding begins four days after birth and peaks at seven to eighteen days before declining after three weeks^12^. During diarrhea episodes, oocyst shedding is typically increased^13^.

Clinically, the age, immunological, and nutritional state of animals can be used to predict how severe cryptosporidiosis will be^14^. Based on data of previous reports, the *Cryptosporidium* prevalence in cattle varies over the world and ranges from 6.25 to 39.65%^15,16^. Cryptosporidiosis prevalence is affected by a variety of factors, including age, hygiene, bedding type, colostrum feeding, herd management, food and water sources, diarrhea, and climate^17^.

Although insensitive, time-consuming, and requiring skilled personnel to detect the organism, the outdated direct microscopic diagnosis of *Cryptosporidium* from faecal samples using acid-fast stain is still as gold standard in many laboratories around the world^18–21^. Only a few studies on animals have employed microscopic methods^22–24^, however some have also used molecular methods^23,25–29^. However, there are few epidemiological data and no risk factor analyses for calve cryptosporidiosis in Egypt. The prevalence of *Cryptosporidium* among ruminant in Ismalia governorates was 32.7% based on PCR assay^30^, 14.19% among buffaloes calves raising in Dakhalia and Kafr Elsheikh governorates using microscopic examination^24^.

The purpose of this study was to estimate the prevalence and assess the associated risk factors for *Cryptosporidium* infection in newborn calve in three governorates situated at Nile Delta of Egypt.

## Materials and methods

### Ethical statement

The ethical committee for animal research at Benha University approved the entire study's methodology and procedures. Informed consent was obtained from owners of examined claves. The Faculty of Veterinary Medicine's ethical committee ensured that all procedures were carried out in accordance with the relevant laws and guidelines. The ARRIVE criteria were followed during research procedure.

### Study area

**Figure 1:**
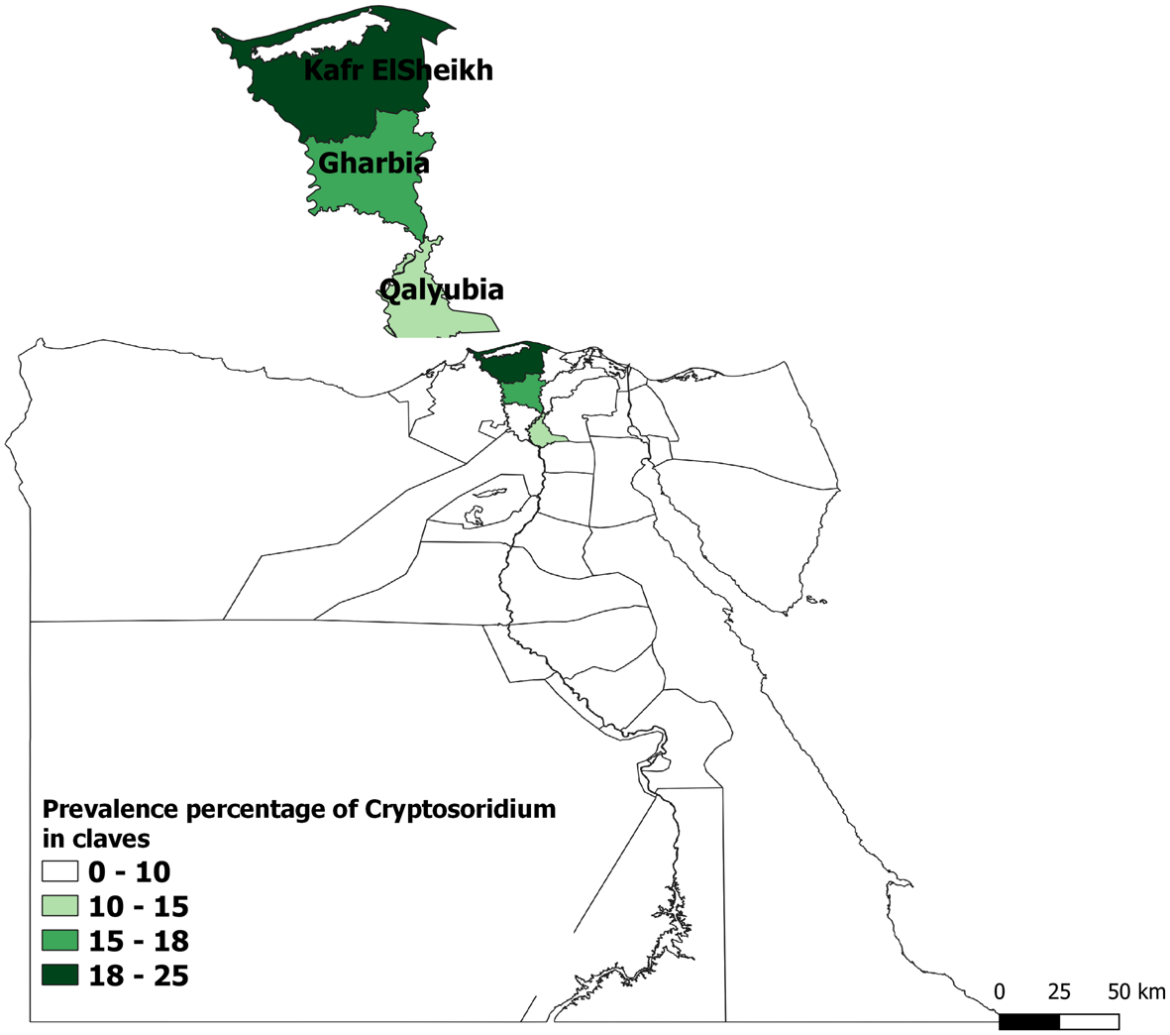
Prevalence of *Cryptosporidium* in calves in different studied areas (MAP generated by QGIS software, https://qgis.org/).

The study was performed in three governorates situated at Nile Delta of Egypt. The governorates selected for the study are Kafer ElSheikh, Qalyubia, and Gharbia, which are located in latitudes of 31° 06′ 42′′ N, 30.41° N, and 30.867° N, respectively, and longitudes of 30° 56′ 45′′ E, 31.21° E, and 31.028° E, Fig. 1.

The Delta is the driest region in the country, has relatively mild temperatures, which increase to 38 °C in summer season. On average, the delta receives 100–200 mm of rain each year, with the majority of this falling during the winter months. These research areas focus mostly on agriculture, livestock husbandry, and have a high number of farms and pastures.

### Sample size and sampling

A cross sectional study was conducted during October 2020 to September 2021 using simple random sampling approach to achieve the forementioned goals. Based on Thrusfield's formula^31^, the sample size was calculated using an expected prevalence of 19.2%^30^ at a 95% confidence interval and a 5% precision value. Consequently, 238 cow calves were included in the sample. However, 430 cow calves in total were enlisted to gather the necessary faeces samples. Using sterile plastic gloves, each calf's individual faeces was collected directly from the rectum and preserved at 4 °C before being transported to the laboratory.

### Questionnaire

At the time of sampling, the farmer completed the provided questionnaire, which mostly asked about animal-related information including breed, age, sex, and body condition. Data on management, including feed source (pasture and milk or pasture), hygienic status (good or poor), the presence of diarrhea (yes or no), and contact with other domestic animals (yes or no), were also gathered.

### Sample analysis

**Figure 2:**
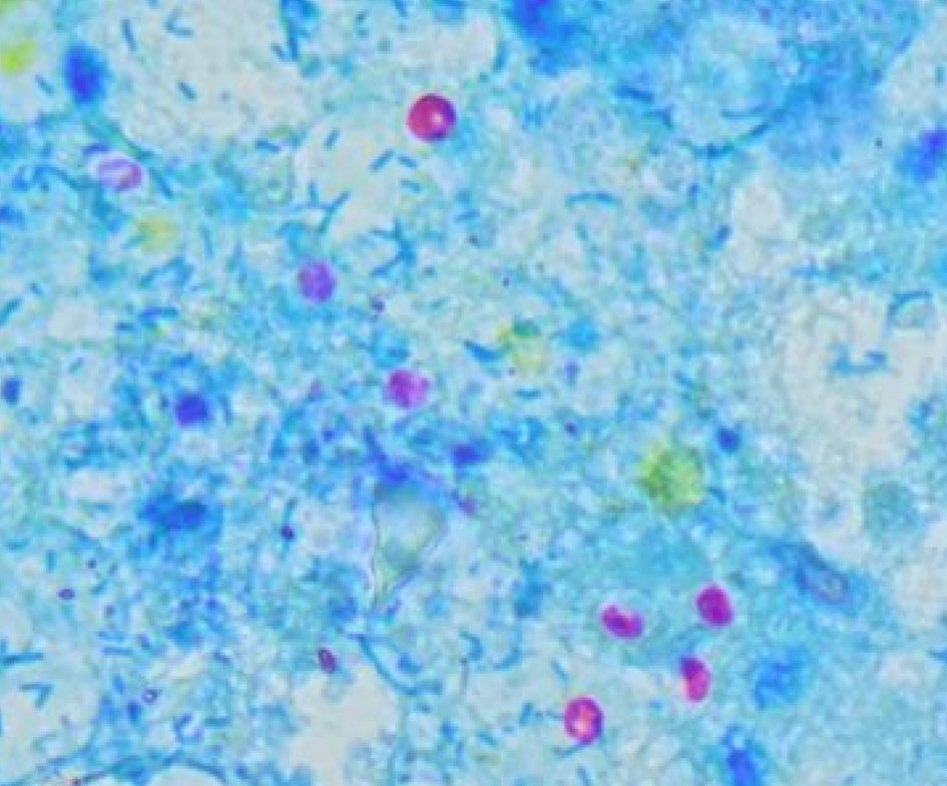
Microscopy of *Cryptosporidium* oocysts in diarrheal calves' faeces stained with Ziehl–Neelsen stain (×1000).

The samples were transferred to the laboratory for further processing on the same day they were collected. After that, the samples were treated via faecal flotation in a Sheather's sugar solution^24^. Then, fecal smear was prepared and stained by modified Ziehl–Neelsen stain^32^. According to Anderson^33^, the severity of the infection was determined by counting the cryptosporidial oocysts in a field at a 1000× magnification; the levels were mild (1–5 oocysts/field), moderate (6–20 oocysts/field), and severe (more than 20 oocysts/field), Fig. 2.

### Molecular identification of cryptosporidiosis

The QIAamp DNA stool Mini Kit (QIAGEN, Hilden, Germany) was used to extract direct DNA from 220 mg of faeces samples according to the manufacturer's instructions. The *Cryptosporidium* small subunit (18S) rRNA gene was amplified with the oligonucleotide primers CRP-DIAG1 forward (5-TTCTAGAGCTAATACATGCG-30) and CRP-DIAG1 reverse (50-CATTTCCTTCGAAACAGGA-30). The PCR assay was performed in total volume of 25 µl including 1 µl of each primer of (20 pmol), 12.5 µl of PCR Master Mix (EmeraldAmp Max, Takara, Japan), 5.5 µl of water, and 5 µl of DNA template. The PCR Conditions was performed as described by Paul et al.^34^.

For the secondary/nested PCR, 1 µl of the primary PCR products was utilized as a template and amplified using the primers CRP-DIAG2 forward (50-GGAAGGGTTATTTATTAGATAAAG-30) and CRP-DIAG2 reverse (50-AAGGAGTAAGGAACAACCTCCA-30). The reaction mixture was initially denaturated at 94 °C for 5 min, followed by 35 cycles of denaturation at 94 °C for one min, annealing at 57 °C for 1 min, elongation at 72 °C for 1 min, and final elongation at 72 °C for 10 min as described by Paul et al.^34^. The amplified products were identified using 1.5% agarose gel electrophoresis and ethidium bromide staining.

### Data analysis

The statistical SPSS software programme, Version 24.0 (IBM, USA), was used for all data analyses. To ascertain the relationship between predicted risk variables and the occurrence of *Cryptosporidium* infection, the univariate logistic regression approach was applied. When the *P* value is less than 0.05, the findings are considered statistically significant. A multivariable analysis comprised factors that were significantly (*P* < 0.05) related to the outcome variable in the univariable analysis^20,35–40^. A test for multicollinearity was also conducted to determine confounding factors and assess the fit of the multivariate model.

## Results

**Figure 3:**
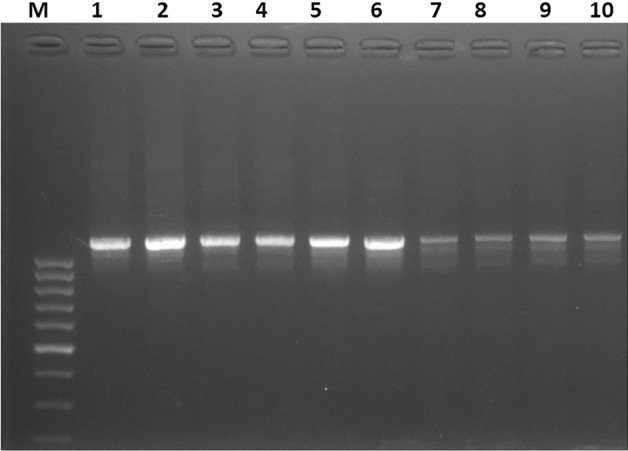
Identification of *Cryptosporidium* spp. using PCR assay targeting 18S rRNA. Lane M: ladder (100 bp) and lane 1–10 positive samples with detected band at 1325bp.

**Figure 4:**
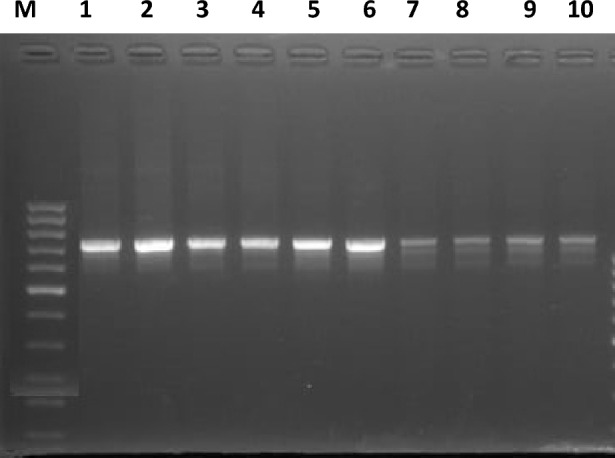
Identification of *Cryptosporidium* *parvum* using PCR assay targeting 18S rRNA. Lane M: ladder (100 bp) and lane 1–10 positive samples for *C. parvum* with detected band at 835 bp.

*Cryptosporidium* oocysts were detected in 81 (18.84%) of the 430 examined calves and the highest prevalence rate (24.67%) was observed in Kafr ElSheikh governorate, but the lowest rate (14.29%) found in Qalyubia governorate, Fig. 1. PCR analysis of all positive samples with microscopic examination revealed predicted bands for *Cryptosporidium* spp. at 1,325 (Fig. 3) in primary PCR which confirm presence of *C. parvum* in all samples in secondary PCR at 835 bp (Fig. 4).

By using univariate logistic regression analysis, five variables had a substantial impact on the prevalence of *Cryptosporidium* infection in calves. The prevalence of the disease was similar between the sex (*P* = 0.764), between breeds (*P* = 0.076), across geographic regions (*P* = 0.064), and in terms of body condition (*P* = 0.785), Table 1.

**Table 1:** The prevalence of *Cryptosporidium* infection in calves in relation to different factors.

Factors	No of examined animals	No of positive	No of negative	% of positive	95% CI	Statistic
Locality						
Kafr ElSheikh	150	37	113	24.67	18.46–32.14	χ2 = 5.494 df = 2 *P* = 0.064
Qalyubia	140	20	120	14.29	9.45–21.04	
Gharbia	140	24	116	17.14	11.8–24.24	
Breed						
Holstein	170	25	145	14.71	10.17–20.81	χ2 = 3.139 df = 1 *P* = 0.076
Mixed-breed	260	56	204	21.54	16.98–26.93	
Sex						
Male	190	37	153	19.47	14.47–25.68	χ2 = 0.090 df = 1 *P* = 0.764
Female	240	44	196	18.33	13.95–23.71	
Age (month)						
< 2	100	13	87	13.00	7.76–20.98	χ2 = 7.905 df = 2 *P* = 0.011*
2–6	150	23	127	15.33	10.44–21.95	
> 6	180	45	135	25.00	19.24–31.8	
Body condition score						
Poor	180	35	145	19.44	14.32–25.83	χ2 = 0.075 df = 1 *P* = 0.785
Good	250	46	204	18.40	14.09–23.67	
Feed source						
Pasture	150	18	132	12.00	7.73–18.17	χ2 = 7.043 df = 1 *P* = 0.008*
Pasture and milk	280	63	217	22.50	18–27.74	
Hygienic status						
Good	170	22	148	12.94	8.7–18.82	χ2 = 6.393 df = 1 *P* = 0.011*
Poor	260	59	201	22.69	18.02–28.16	
Presence of diarrhea						
Yes	320	70	250	21.88	17.7–27.63	χ2 = 7.550 df = 1 *P* = 0.006*
No	110	11	99	10.00	5.68–17.02	
Contact with domestic animals						
Yes	190	48	142	25.26	19.61–31.89	χ2 = 9.194 df = 1 *P* = 0.002*
No	240	33	207	13.75	9.96–18.68	
Total	430	81	349	18.84	15.43–22.81	

*The result considered significant if *P* < 0.05.

The prevalence in calves older than six months was substantially (*P* = 0.011) greater than in calves younger than six months. In addition, the *Cryptosporidium* infection increased significantly in calves living in poor hygienic condition (22.69%, 95%CI 18.02–28.16) compared to calves living in good condition status (12.94%, 95%CI 8.7–18.82), and it was significantly higher in calves feeding on pasture and milk (22.5%, 95%CI 18–27.74) than in calves feeding on pastures only (12%, 95%CI 7.73–18.17), Table 1. Additionally, compared to non-diarrheic calves, diarrheic calves had a considerably higher prevalence of *Cryptosporidium* (21.88%, 95% CI 17.7–27.63, *P* = 0.006), and calves that had contact with other domestic animals had a significantly higher prevalence (25.26%, 95% CI 19.61–31.89, *P* = 0.002), Table 1.

Table 2 showed the results of multivariate logistic regression analysis on significant factors (*P* < 0.05) in univariate analysis, which were age, feed source, sanitary state, presence of diarrhea, and contact with other domestic animals. The prevalence of *Cryptosporidium* infection increased significantly with age, older calves were two times (OR 1.96, 95%CI 0.97–3.94) more likely to contract the *Cryptosporidium* infection as compared to young calves. Farms had poor hygiene condition and pasture and milk as source of feeding increased the risk of *Cryptosporidium* infection by two folds (OR 2.25, 95%CI 1.28–3.94) and (OR 2.07, 95%CI 1.15–3.72), respectively. Animals with diarrhea were 2.47 times (OR 2.47, 95%CI 1.23–4.96) more likely to acquire *Cryptosporidium* infection than normal calves. Moreover, the risk of *Cryptosporidium* infection increased two times (OR 2.08, 95%CI 1.24–3.50) more among calves in contact with domestic animals than other.

**Table 2:** Risk factors associated with *Cryptosporidium* prevalence in calves.

Variable	B	S.E	OR	95% CI for OR	*P* value
Age					
2–6	0.178	0.385	1.19	0.56–2.54	0.645
> 6	0.672	0.356	1.96	0.97–3.94	0.039
Feed source					
Pasture and milk	0.727	0.300	2.07	1.15–3.72	0.015
Hygienic status					
Poor	0.810	0.287	2.25	1.28–3.94	0.005
Presence of diarrhea					
Yes	0.906	0.355	2.47	1.23–4.96	0.011
Contact with domestic animals					
Yes	0.732	0.265	2.08	1.24–3.50	0.006

*B* Logistic regression coefficient, *SE* Standard error, *OR* Odds ratio, *CI* Confidence interval.

## Discussion

Cryptosporidiosis in animals is considered a zoonotic risk to humans, due to the release of large numbers of resistant oocysts, which contaminate surface water. The Veterinary researchers were interested in cryptosporidiosis because, in addition to its zoonotic significance, it may develop into a dangerous, difficult-to-control disease in many farm animals and cause large economic losses. The present study aimed to evaluate the prevalence of *Cryptosporidium* infection and asses the associated risk factors in calves.

In the present study, the total *Cryptosporidium* prevalence in calves was found to be 18.84% (81/430). This corresponds to the findings of Ayele et al.^41^, who reported an infection rate of 18.6% in dairy calves in northwest Ethiopia. In addition, the prevalence rate in this study is consistent with the previously reported rate (19.2%) for bovine calves in Ismailia governorates in Egypt^30^. Similar prevalence rate (17.9%) was found in dairy calves from France^42^. This study's prevalence rate for *Cryptosporidium* infection was lower than the reported rates in eastern Ethiopia 27.8% by Regassa et al.^43^, USA 35.5% by Santın et al.^44^, Vietnam 44.3% by Nguyen et al.^45^ and in UK 27.9% by Brook et al.^46^ but higher than 7.8% in, 13.6%, and 15.8% which reported by Wegayehu et al.^47^, Ayana and Alemu^48^, and Wegayehu et al.^49^ in Ethiopia, respectively.

Furthermore, the detectable species in examined calves was *C. parvum* which come in accordance with the findings of Singh et al.^50^ who reported 79.41% of young dairy calves in Punjab infected by *C. parvum*. Also, other studies reported the more prevalent *Cryptosporidium* species in calves in Ethiopia and Egypt is *C. parvum* with prevalence rate of 18.6% and 32.2%, respectively^30,51^.

The differences in overall *Cryptosporidium* prevalence between studies could be attributed to differences in ecology, research design, seasons, management system, age, herd size, and laboratory tests used^23,28,46,48,52–58^. The diagnostic procedures used in this survey are less sensitive and can produce misleading negative results. This could potentially be the explanation for report variation^59^.

The sex had no effect on the prevalence of *Cryptosporidium* infection in the current study, which come in agreement with previous findings of Paul et al.^34^. In contrast, other studies reported significant role for sex on prevalence of *Cryptosporidium* in calves^32,60^.

The significant effect of age on *Cryptosporidium* prevalence in calve in this study was consistent with previous findings of Wegayehu et al.^49^in Ethiopia, they found higher prevalence in calves older than 3 months and stated infection was age related and 92.1% were infected with *C. andrsoni* which infect older age calves. In contrast, Geurden et al.^61^, Ayele et al.^41^ and Venu et al.^59^ stated that infection rate decreased with the increase of age. Similarly, the effect of age on prevalence of *Cryptosporidium* infection in calve was observed in other studies^16,41^. This might be due to lower tolerance of young calves as a result of immature immune system. Calves under four months of age are more susceptible to contracting *Cryptosporidium* infection^46^. This findings is consistent with the findings of Kváč et al.^62^, who observed that infection resistance can evolve with age due to immunological development over time.

Additionally, a significant correlation was found between the hygienic condition of the farm and the occurrence of *Cryptosporidium* infection in calves. The current result is confirmed by the findings of Abebe et al.^63^, who found a significant association between *Cryptosporidium* infection and the hygiene status of the farms. In addition, a similar results were reported by El-Khodery and Osman^24^ and Castro-Hermida et al.^64^, they confirmed that poor hygiene enhances the infection rate and dissemination of *Cryptosporidium* species in animals. This could be attributed to the fact that muddy or dirty farm could probably establish a favourable condition for the presence or existence of *Cryptosporidium* oocysts in the surround environment of animals. This can be increasing the risk of contamination of food and water, hence increase the risk of *Cryptosporidium* infection in calves^41,52,65–70^.

The present findings are directly in line with previous findings of Ayele et al.^41^, who observed that the prevalence of *Cryptosporidium* increased significantly among calves feeding on pasture and milk. This may be due to pasture being contaminated with infectious oocysts, and switching between pasture and milk may produce digestive disturbances that increase the prevalence of cryptosporidiosis.

Cryptosporidiosis causes sever watery diarrhea in calves. The findings of the present study revealed strong association between presence of diarrhea and prevalence of *Cryptosporidium* in calves. This was explained by the fact that diarrheal animals had a higher rate of oocyst shedding than calves with regular faeces. This is consistent with those has been found in previous studies of El-Khodery and Osman^24^ and Abebe et al.^63^. This could be as a result of the infection causing villous atrophy and crypt hyperplasia, which reduces the intestine's surface area available for absorption^71^. Consequently, interfere with absorption of water, glucose and sodium leading to diarrhea^72^. Additionally, the parasite may be able to decrease the activity of the enzyme disaccharidase, which would reduce the amount of sugars broken down. This would lead to bacterial growth, the production of volatile fatty acids, and changes in osmotic pressure, which would then cause severe watery diarrhea^73^.

Different animal species and human are susceptible to *Cryptosporidium* infection and the infection can be transmitted by direct or indirect routes through fecal–oral route^32^. Consequently, mixing different animals species could facilitate the transmission of the disease^42^. Similarly, Mohammed et al.^74^ observed that keeping animals separately or avoiding close contact with animals of various species can reduce the risk of *Cryptosporidium* infection.

## Conclusion

The prevalence of *Cryptosporidium* infection was widely spreading among calves in the studied governorates with rate of 18.84%. Based on the present findings, age, feed source, farm hygiene, occurrence of diarrhea, and interaction with various domestic animals were all risk factors for *Cryptosporidium* infection. It is essential to raise awareness of risk factors, sources of infection, and modes of transmission to control and reduce the disease transmission between animals and human. Further molecular researches in different areas of the country are recommended to determine species allocation and the disease's national impact.

### Supplementary Information

Supplementary Information 1.

## Data Availability

All data generated or analysed during this study are included in this published article.
