# Supplementary figures and images for: Prevalence of Cryptosporidium infection and associated risk factors in calves in Egypt
Source: Sci Rep. 2023 Oct 18;13:17755. doi: 10.1038/s41598-023-44434-7 (PMC10584872; doi:10.1038/s41598-023-44434-7)

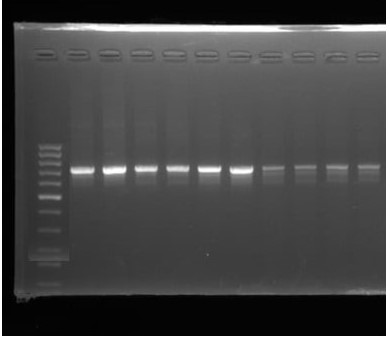

Supplement: Supplementary file 1 — Supplementary Information 1. [file 41598_2023_44434_MOESM1_ESM.jpg]
